# Evolutionary game analysis of multiple subjects in the management of major public health emergencies

**DOI:** 10.1016/j.heliyon.2024.e29823

**Published:** 2024-04-17

**Authors:** Rui Nan, Jing Chen, Wenjun Zhu

**Affiliations:** School of Law and Humanities, China University of Mining and Technology (Beijing), Beijing, 100083, China

**Keywords:** Public health emergency, Emergency management, Multiple subjects, Evolutionary game

## Abstract

The frequent occurrence of major public health emergencies (MPHEs) significantly challenges national security, economic stability, social operation and the safety of people's lives and property worldwide. Consequently, enhancing the emergency management of MPHEs is critically urgent. This paper constructs a game model involving local government, social organisations, and the public for MPHE management, exploring strategy combinations and influencing factors across various scenarios. Several results were obtained. (1) Local government, social organisations, and the public each have positive and negative strategy choices based on cost–benefit analysis, leading to eight different strategy combinations. Furthermore, all three take positive strategies as the optimal way to achieve the game equilibrium. (2) The transformation of strategy combinations is primarily influenced by the cost-benefit gap and the strategic decisions of local government. (3) Altering a subject's initial strategy value doesn't change its final choice but impacts the time to achieve a stable strategy equilibrium. The severity of local government punishments on social organisations influences their strategic choices and the time to optimal strategy, whereas rewards to the public or social organisations only affect the time to achieve this strategy. The findings of this study can not only help improve the collaborative governance system of MPHEs but also provide scientific guidance on how governments can manage MPHEs.

## 1 Introduction

Major public health emergencies (MPHEs), such as the spread of severe infectious diseases, are an important type of public safety events. Compared with natural disasters, accidents, and social security incidents, MPHEs tend to spread intermittently and extensively, facilitated by human and logistics movements, resulting in rapid cross-regional diffusion. In recent years, MPHEs have become more frequent, complex, and difficult to prevent and control [1]. For example, the COVID-19 outbreak in early 2020, a highly contagious and widespread MPHE, significantly impacted public health, economic development, and social stability. This pandemic rigorously evaluated the resilience and efficacy of global governance frameworks and national capacities. Consequently, strengthening capacities regarding the emergency management of MPHEs and effectively controlling their catastrophic effects have become issues of great concern to all countries [2]. In response, some scholars have conducted objective analyses of infectious disease characteristics using the SEIR (susceptible-exposed-infected-susceptible) and SEAIR (susceptible-exposed-asymptomatic-infected-recovered) models from a technological standpoint [3,4]. Additionally, researchers have evaluated emergency responses by integrating mobile device data with infectious disease models [5].

The abovementioned approaches can help reduce infections in the affected populations and set scientific pathways and targets for the optimal allocation of healthcare resources [6,7]. However, the increase in productivity brought about by technological innovation has rendered traditional emergency management systems obsolete. Thus, innovating crisis management systems is essential to enhance resource allocation efficiency and achieve Pareto optimization in social resource distribution. This raises a crucial issue: how to effectively coordinate and allocate emergency resources [8]. Considering these developments, there is a discernible pivot in the academic community from technical proficiencies to an emphasis on strategic management. This involves leveraging innovative crisis management systems to establish efficient multisubject cooperation mechanisms for optimal emergency resource coordination and distribution [9].

Given the growing complexity of MPHEs, local governments, social organisations, the public, and other individual entities alone cannot efficiently manage emergencies. In recent years, collaborative governance of multiple subjects has become the main practice to realise the effective management of MPHEs through orderly collective action. Therefore, a scientific understanding of the behavioural strategies of multiple subjects in MPHE and their evolution, along with the construction of a multiple-subject collaboration mechanism to optimize the allocation of emergency response resources and promote the modernisation of emergency management capabilities, have become key issues in the emergency management of MPHEs.

Academic research on the management of MPHEs is based on two perspectives. The first one is the static perspective, which focuses on the composition of the main subject of action in MPHE and its strategy selection. Academics usually regard the government, the public, the community, and the media as the game subjects. The government and the public are the important action subjects in MPHEs, the community constitutes the micro field of analysis and social media induces the so-called gas pedal effect. Specifically, governments, as central authorities for MPHEs, faced misalignment and lack of coordination during the COVID-19 crisis, leading to poor economic outcomes, inefficiency, and rigidity [10,11]. Governments are tasked with balancing epidemic control against economic impacts [12]. They should also be able to (1) implement emergency measures, such as blockades and quarantines, to limit the spread of the epidemic in the early stages of the outbreak and protect lives and (2) introduce awareness-raising programs in the later stages of the epidemic to reduce the presence of unprotected susceptible individuals and maintain socioeconomic development [13,14]. At the same time, reasonable policies for subsidies and penalties should be developed [15].

The spread of public health events is also affected by the public's willingness to adopt preventive public health behaviours [16]. Furthermore, the public is affected by a combination of external information triggers and internal psychological perceptions to adopt differential behaviours towards government-promoted measures, such as wearing masks [17,18]. However, such measures have sometimes led to widespread panic buying, further destabilizing social order [19].

Acting as the fourth estate, the media plays a crucial role in information dissemination during MPHEs. While unregulated media may pose health risks [20], regulated media supports governments and emergency agencies in gauging public information needs, fostering public opinion, providing emotional comfort [21], and encouraging public participation [22].

Finally, as an important force in national governance, the community can address government and market failure and play an important role in the control and response to COVID-19 [23,24]. For example, the synergy amongst different principals within the community allowed the community to play an important role in responding to the COVID-19 incident [25].

The second approach in MPHE management research adopts a dynamic perspective, concentrating on the evolution of multiple actors involved in MPHE. Scholars vary in their selection of game subjects, differing in both the number and the nature of these subjects. For example, some scholars focus on official agencies and disaster relief organisations in exploring how dual subjects decide ‘which rumour to clarify’ and ‘when to clarify’ by constructing two sequential game models: ‘rumour selection clarification’ and ‘learning to clarify rumour’. This creation of sequential game models enables a detailed examination of how these entities choose 'which rumour to clarify' and 'when', enhancing understanding of their decision-making processes [26].

Meanwhile, some scholars have focused on government agencies, the Internet media, and the public, applying the three-way evolutionary game model and system dynamics approach to explore the heterogeneous effects of different initial strategies and epidemic transmission probabilities on the strategy evolution of the triadic subjects in COVID-19 [27]. Additionally, researchers have differentiated between central and local governments to investigate the development of joint prevention and control mechanisms involving both government levels and NGOs within urban markets [28]. Other scholars focused on the relationships between governments, enterprises, and consumers, creating three-way evolutionary game models to determine whether the penalty mechanism is more effective than the subsidy mechanism in achieving the COVID-19 joint prevention goal [29]. Some researchers have explored the links between government, social organisations and the public to explore the evolutionary game of the emergency social mobilisation network for major emergencies [30].

While many studies have used tripartite evolutionary game models to examine strategy evolution in MPHEs, they often overlook social organisations, which are crucial in bridging the government and the public. Additionally, comprehensive analyses encompassing the local government, the public, and social organisations are scarce. To make up for this shortcoming, the present study not only constructs a triadic subjective game analysis model, thus benefiting the matrix of local government, social organisations, and the public in MPHEs, but also carries out a specific analysis of eight different strategy combinations. It also examines the dynamics of cost-benefit trends and the impact of local government actions on equilibrium strategies using simulation. At the same time, it further discusses the influence mechanism of the change of the initial value of different subject strategies and the strength of local government rewards and punishments on the evolution of each subject strategy. This approach enhances collaborative governance systems for MPHEs and offers a scientific foundation to guide government actions during such emergencies.

In the context of MPHEs, due to the actions of multiple subjects interacting with one another and easily suffering from the constraints of the external environment and social dilemma [31,32], the objectives of a multiple-subject approach are not completely consistent and often take the game behaviour to play with each other. During the emergency management process, multiple subjects will go through repeated game processes and the subjects involved in the game will gradually form interest-organising groups in their repetitive behaviours. This dynamic can lead to corrupt and transactional behaviours [33], complicating the achievement of an equilibrium strategy. Game models effectively capture the influence and interdependence among decision-makers [34], proving especially useful in developing cooperative mechanisms under complexity. By elucidating interests and influencing behaviours, game models clarify subjects' objectives and guide their decisions. This can be achieved by using the evolutionary game method to construct a game model of multiple subjects, such as local government, social organisations, and the public during MPHEs, as well as exploring the strategic combinations of multiple subjects and their influencing factors in different contexts. This approach not only clarifies actors’ interests but also steers their decisions towards maximizing social and public benefits, facilitating rapid development of a collaborative equilibrium strategy and effective collective action in MPHE [35].

The paper continues as follows: Section 2 introduces the research hypotheses and develops a multisubject game model. Section 3 discusses the results from numerical simulations in detail. Section 4 summarises the main conclusion and offers further discussion on the topic.

## 2 Model construction

### 2.1 Research hypothesis

In the emergency management of MPHEs, the evolutionary game of multiple subjects follows three principles, the first of which is the principle of efficacy maximisation. The subjects (local government, social organisations, and the public), viewed as ‘economic entities’ are driven by their interests. Thus, they opt for strategies that align with their interests, considering each other's choices and the benefits thereof to enhance mutual understanding. The synergy amongst the three subjects under the evolution of different scenarios results in the maximisation of MPHE effectiveness.

The second principle, behavioural modification, and long-term goals, dictates that each subject adjusts its strategy over time, considering long-term objectives, costs, and evolving concepts, ultimately selecting more scientifically grounded approaches. The third principle, evolutionary game equilibrium, involves each subject maximizing its interests through persistent efforts and tacit coordination until an evolutionary equilibrium is reached, fostering governance synergy. Following these principles, Table 1 details the symbols and meanings of parameters in Hypotheses 1–5.

Hypothesis 1 In the multisubject game of MPHE, all three subjects have limited rationality, and the local government can adopt either the ‘positive governance’ or ‘negative governance’ strategy, social organisations can adopt the ‘positive organisation’ or ‘negative organisation’ strategy, and the public can adopt the ‘positive participation’ or ‘negative participation’ strategy.

Hypothesis 2 The probabilities of a local government adopting the ‘positive governance’ and ‘negative governance’ strategies are x and 1‐x (0≤x≤1), respectively; the probabilities of social organisations adopting ‘positive organisation’ and ‘negative organisation’ strategies are y and 1‐y (0≤y≤1), respectively; and the probabilities of the public adopting ‘positive participation’ and ‘negative participation’ strategies are z and 1‐z (0≤z≤1), respectively.

Hypothesis 3 In MPHE, the cost of a local government's positive governance strategy is $C_{g1}$, which mainly includes the human capital mobilised by the local government to participate in the emergency response, the cost of medical supplies required for the emergency response, and the cost of life support needed to ensure the stable and orderly operation of society. Meanwhile, the social benefit is $R_{g1}$. The cost of the local government's negative governance strategy is $C_{g2}$, which only includes the necessary governance costs, and the social benefit currently is $R_{g2}$. In addition, because the government's negative governance strategy will bring a series of adverse effects, it is difficult to obtain social recognition and even provokes public dissatisfaction, resulting in a loss of the public trust $T_{g}$, where $C_{g1}>C_{g2}$, $R_{g1}>R_{g2}$.

Hypothesis 4 When social organisations adopt the positive organisation strategy to participate in social mobilisation and utilise their professional capabilities, the cost is $C_{s1}$ and the social benefit is $R_{s1}$. Meanwhile, due to social organisations playing an important role in the management of MPHE, the local government will reward its positive strategy $V_{s}$. When social organisations adopt the negative organisation strategy, which is to sacrifice public interests to satisfy their interests in the process of emergency management, the cost is $C_{s2}$ and the social benefit is $R_{s2}$. At the same time, the social benefit is the general benefit after behaviour correction. When its negative strategy has a large adverse impact on society, the local government will intervene in its behaviour and take punishment $F_{s}$. In addition, it also brings reputation loss $T_{s}$, where $C_{s1}>C_{s2}$, $R_{s1}>R_{s2}$.

Hypothesis 5 In MPHE, when the public adopts the positive participation strategy to participate effectively in the grassroots emergency by cooperating with local government, the cost is $C_{p1}$, which includes the opportunity cost caused by the investment of time and resources. Positive participation brings satisfaction, and the benefit is $R_{p1}$. At the same time, the government will give the public reward of $W_{p}$. When the public adopts the negative participation strategy, it needs to bear the loss of poor governance The cost is $C_{p2}$ and the social benefit is $R_{p2}$, where $C_{p1}>C_{p2}$, $R_{p1}>R_{p2}$.

**Table 1:** Parameter symbols and meanings of local government, social organisations, and the public.

Subjects	Parameter	Meaning	Note
**Local Government**	$C_{g1}$	The cost of the ‘positive governance’ strategy by the local government	$C_{g1}>C_{g2}$ $R_{g1}>R_{g2}$
	$C_{g2}$	The cost of the ‘negative governance’ strategy by the local government	
	$R_{g1}$	The social benefit of the ‘positive governance’ strategy by the local government	
	$R_{g2}$	The social benefit of the ‘negative governance’ strategy by the local government	
	$T_{g}$	The loss of public trust with the ‘negative governance’ strategy for the local government	
**Social Organisations**	$C_{s1}$	The cost of the ‘positive organisation’ strategy by social organisations	$C_{s1}>C_{s2}$ $R_{s1}>R_{s2}$
	$C_{s2}$	The cost of the ‘negative organisation’ strategy by social organisations	
	$R_{s1}$	The social benefit of the ‘positive organisation’ strategy by social organisations	
	$R_{s2}$	The social benefit of the ‘negative organisation’ strategy by social organisations	
	$V_{s}$	Local government rewards for social organisations' positive organisation strategy	
	$F_{s}$	Local government punishment for social organisations' negative organisation strategy	
	$T_{S}$	The reputational loss with the ‘negative organisation’ strategy for social organisations	
**The Public**	$C_{p1}$	The cost of the ‘positive participation’ strategy by the public	$C_{p1}>C_{p2}$ $R_{p1}>R_{p2}$
	$C_{p2}$	The cost of the ‘negative participation’ strategy by the public	
	$R_{p1}$	The social benefit of the ‘positive participation’ strategy by the public	
	$R_{p2}$	The social benefit of the ‘negative participation’ strategy by the public	
	$W_{p}$	Local government reward for the public's positive participation strategy	

### 2.2 Model analysis

Based on the above hypotheses, the costs involved when the local government, social organisations and the public adopt the positive strategy are higher than those for the negative strategy; furthermore, the social benefit of the former is higher than the latter. When the local government adopts the positive governance strategy, it rewards social organisations and the public for their positive strategies; when social organisations adopt the negative organisation strategy and bring harmful effects to society, it will not only bring a negative reputation to themselves and reduce their credibility but also risk being punished by the local government. By setting model parameters, the benefits matrix for the local government, social organisations, and the public is defined through strategy combinations and game relationships, as detailed in Table 2.

**Table 2:** Three subjects game benefit matrix of local government, social organisations, and the public.

		Social organisations	The public	
			Positive participation（z）	Negative participation（1‐z）
Local government	Positive governance （x）	Positive organization（y）	$R_{g1}‐C_{g1}‐V_{s}‐W_{p}$ $R_{s1}‐C_{s1}+V_{s}$ $R_{p1}‐C_{p1}+W_{p}$	$R_{g1}‐C_{g1}‐V_{s}$ $R_{s1}‐C_{s1}+V_{s}$ $R_{p2}‐C_{p2}$
		Negative organization（1‐y）	$R_{g1}‐C_{g1}+F_{s}‐W_{p}$ $R_{s2}‐C_{s2}{‐F}_{s‐}T_{s}$ $R_{p1}‐C_{p1}+W_{p}$	$R_{g1}‐C_{g1}+F_{s}$ $R_{s2}‐C_{s2}{‐F}_{s‐}T_{s}$ $R_{p2}‐C_{p2}$
	Negative governance （1‐x）	Positive organization（y）	$R_{g2}‐C_{g2}‐T_{g}$ $R_{s1}‐C_{s1}$ $R_{p1}‐C_{p1}$	$R_{g2}‐C_{g2}‐T_{g}$ $R_{s1}‐C_{s1}$ $R_{p2}‐C_{p2}$
		Negative organization（1‐y）	$R_{g2}‐C_{g2}‐T_{g}$ $R_{s2}‐C_{s2}‐T_{s}$ $R_{p1}‐C_{p1}$	$R_{g2}‐C_{g2}‐T_{g}$ $R_{s2}‐C_{s2}‐T_{s}$ $R_{p2}‐C_{p2}$

#### 2.2.1 Multisubject replication dynamic analysis

(1) Replication dynamic equations for local government strategies

The expected and average benefits for local government adopting ‘positive governance’ and ‘negative governance’ strategies are respectively given by:

$${\;U}_{g1}=yz\left(R_{g1}‐C_{g1}‐V_{s}‐W_{p}\right)+\left(1‐y\right)z\left(R_{g1}‐C_{g1}+F_{s}‐W_{p}\right)$$

$$+y\left(1‐z\right)\left(R_{g1}‐C_{g1}‐V_{s}\right)+\left(1‐y\right)\left(1‐z\right)\left(R_{g1}‐C_{g1}+F_{s}\right)$$

(1) $$=R_{g1}‐C_{g1}‐yV_{s}‐zW_{p}+\left(1‐y\right)F_{s}$$

$${\;U}_{g2}=yz\left(R_{g2}‐C_{g2}‐T_{g}\right)+\left(1‐y\right)z\left(R_{g2}‐C_{g2}‐T_{g}\right)$$

$$+y\left(1‐z\right)\left(R_{g2}‐C_{g2}‐T_{g}\right)+\left(1‐y\right)\left(1‐z\right)\left(R_{g2}‐C_{g2}‐\right)$$

(2) $$=R_{g2}‐C_{g2}‐T_{g}$$

(3) $$\bar{U}=xU_{g1}+\left(1‐x\right)U_{g2}$$

In accordance with the Malthusian model, the evolutionary game replication dynamic equation of local government is expressed as:

$$F\left(x\right)=\frac{d_{x}}{d_{t}}=x\left(U_{g1}‐\bar{U_{g}}\right)=x\left(1‐x\right)\left(U_{g1}‐U_{g2}\right)$$

(4) $$=x\left(1‐x\right)\left[y\left(F_{s}‐V_{s}\right)‐zW_{p}+R_{g1}‐C_{g1}‐R_{g2}+C_{g2}+T_{g}+F_{s}\right]$$

(2) Replication dynamic equations for social organisations strategies

The expected and average benefits for social organisations adopting ‘positive organisation’ and ‘negative organisation’ strategies are respectively expressed as:

$${\;U}_{s1}=xz\left(R_{s1}‐C_{s1}+V_{s}\right)+x\left(1‐z\right)\left(R_{s1}‐C_{s1}+V_{s}\right)$$

$$+\left(1‐x\right)z\left(R_{s1}‐C_{s1}\right)+\left(1‐x\right)\left(1‐z\right)\left(R_{s1}‐C_{s1}\right)$$

(5) $$={xV}_{s}+R_{s1}‐C_{s1}$$

$${\;U}_{s2}=xz\left(R_{s2}‐C_{s2}‐F_{s}{‐T}_{s}\right)+x\left(1‐z\right)\left(R_{s2}‐C_{s2}‐F_{s}‐T_{s}\right)$$

$$+\left(1‐x\right)z\left(R_{s2}‐C_{s2}‐T_{s}\right)+\left(1‐y\right)\left(1‐z\right)\left(R_{s2}‐C_{s2}‐T_{s}\right)$$

(6) $$=‐{xF}_{s}+R_{s2}‐C_{s2}‐T_{s}$$

(7) $$\bar{U_{s}}=yU_{s1}+\left(1‐y\right)U_{s2}$$

Based on the Malthusian model, the evolutionary game replication dynamic equation of social organisations is expressed as:

$$F\left(y\right)=\frac{d_{y}}{d_{t}}=y\left(U_{s1}‐\bar{U_{s}}\right)=y\left(1‐y\right)\left(U_{s1}‐U_{s2}\right)$$

(8) $$=y\left(1‐y\right)\left[x\left(V_{s}+F_{s}\right)+R_{s1}‐C_{s1}‐R_{s2}+C_{s2}+T_{s}\right]$$

(3) Replication dynamic equations for the public strategies

The expected and average benefits for the public adopting ‘positive participation’ and ‘negative participation’ strategies are given by:

$${\;U}_{p1}=xy\left(R_{p1}‐C_{p1}+W_{p}\right)+x\left(1‐y\right)\left(R_{p1}‐C_{p1}+W_{p}\right)$$

$$+\left(1‐x\right)y\left(R_{p1}‐C_{p1}\right)+\left(1‐x\right)\left(1‐y\right)\left(R_{p1}‐C_{p1}\right)$$

(9) $$={xW}_{p}+R_{p1}‐C_{p1}$$

$${\;U}_{p2}=xy\left(R_{p2}‐C_{p2}\right)+x\left(1‐y\right)\left(R_{p2}‐C_{p2}\right)$$

$$+\left(1‐x\right)y\left(R_{p2}‐C_{p2}\right)+\left(1‐x\right)\left(1‐y\right)\left(R_{p2}‐C_{p2}\right)$$

(10) $$=R_{p2}‐C_{p2}$$

(11) $$\bar{U_{p}}=zU_{p1}+\left(1‐z\right)U_{p2}$$

Following the Malthusian model, the evolutionary game replication dynamic equation of the public is given by:

$$F\left(z\right)=\frac{d_{z}}{d_{t}}=z\left(U_{p1}‐\bar{U_{p}}\right)=z\left(1‐z\right)\left(U_{p1}‐U_{p2}\right)$$

(12) $$=z\left(1‐z\right)\left[xW_{P}+R_{p1}‐C_{p1}‐R_{p2}+C_{p2}\right]$$

In summary, according to Equations (1), (10), (11), (12), (2), (3), (4), (5), (6), (7), (8), (9), the three-dimensional replication dynamic system equation of the evolutionary game process with the three subjects involved (Equation (13)) can be obtained as follows:

(13) $$\left\{ \begin{array}{c} F\left(x\right)=\frac{d_{x}}{d_{t}}=x\left(1‐x\right)\left[y\left(F_{s}‐V_{s}\right)‐zW_{p}+R_{g1}‐C_{g1}‐R_{g2}+C_{g2}+T_{g}+F_{s}\right]\\ F\left(y\right)=\frac{d_{y}}{d_{t}}=y\left(1‐y\right)\left[x\left(V_{s}+F_{s}\right)+R_{s1}‐C_{s1}‐R_{s2}+C_{s2}+T_{s}\right]\\ F\left(z\right)=\frac{d_{z}}{d_{t}}=z\left(1‐z\right)\left[xW_{P}+R_{p1}‐C_{p1}‐R_{p2}+C_{p2}\right] \end{array}\right.$$

#### 2.2.2 Multisubject evolutionary stabilisation strategy analysis

(1) Triple-subject evolutionary stabilisation strategy analysis

To find the equilibrium point of the system, in accordance with Equation (13), set:

(14) $$\left\{ \begin{array}{c} F\left(x\right)=\frac{d_{x}}{d_{t}}=0\\ F\left(y\right)=\frac{d_{y}}{d_{t}}=0\\ F\left(z\right)=\frac{d_{z}}{d_{t}}=0 \end{array}\right.$$

Based on Equation (14), the stability strategy of the three subjects can be discussed as follows:

The evolutionary game replication dynamic Equation (4) based on the local government leads to the following:

If $z=\frac{\left[y\left(F_{s}‐V_{s}\right)+R_{g1}‐C_{g1}‐R_{g2}+C_{g2}+T_{g}+F_{s}\right]}{W_{p}}$, set F(x)=0, all values of x are steady states.

If $z<\frac{\left[y\left(F_{s}‐V_{s}\right)+R_{g1}‐C_{g1}‐R_{g2}+C_{g2}+T_{g}+F_{s}\right]}{W_{p}}$, set F(x)=0, x=0or1,

Currently, $\frac{dF\left(x\right)}{d_{x}}|x=0>0$, $\frac{dF\left(x\right)}{d_{x}}|x=1<$ 0, x=1 is the equilibrium point, and the local government's positive governance strategy is the evolutionary equilibrium strategy.

**Fig. 1:**
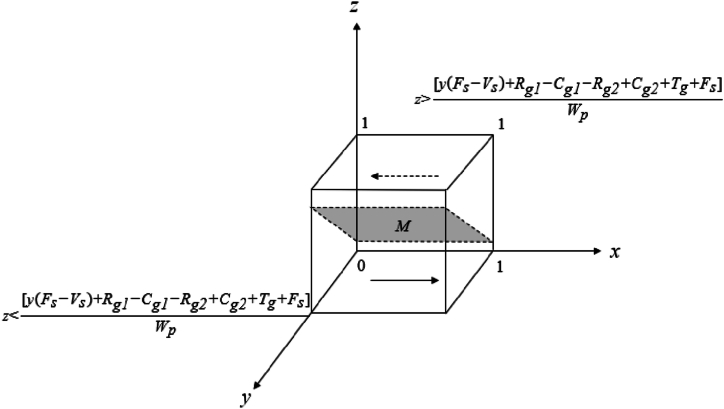
Phase diagram of the evolutionary trend of local government strategies.

If $z>\frac{\left[y\left(F_{s}‐V_{s}\right)+R_{g1}‐C_{g1}‐R_{g2}+C_{g2}+T_{g}+F_{s}\right]}{W_{p}}$, set F(x)=0, x=0or1,

Currently, $\frac{dF\left(x\right)}{d_{x}}|x=0<0$, $\frac{dF\left(x\right)}{d_{x}}|x=1>$ 0, x=0 is the equilibrium point, and the local government's negative governance strategy is the evolutionary equilibrium strategy (Fig. 1).

In Fig. 1, the default in this paper is that local government punishes social organisations' negative organisation strategy more than it rewards their positive organisation strategy to better play the role of government mobilisation. The local government's positive governance strategy is not only related to its benefits and reputation losses but also related to the reward and punishment mechanisms for social organisations and rewards for the public. Assuming other parameters remain constant, the local government's stable strategy options include:

① The local government is more likely to adapt a positive governance strategy when its benefits outweigh its costs, especially if negative governance strategy leads to a significant loss of public trust.
② When the gap between the local government's punishment for negative organisation strategy and the reward for positive organisation strategy is increasing, the deterrence effect of punishment becomes greater than the incentive effect of reward. Furthermore, the local government governance becomes more positive.
③ Increasing rewards for positive public participation boosts motivation, simultaneously encouraging the local government to favour positive governance strategies.

The evolutionary game replication dynamic Equation (8) based on social organisations leads to the following:

If $x=\frac{R_{s2}‐C_{s2}‐R_{s1}+C_{s1}‐T_{s}}{V_{s}+F_{s}}$, F(y)=0, all values of y are steady states.

If $x>\frac{R_{s2}‐C_{s2}‐R_{s1}+C_{s1}‐T_{s}}{V_{s}+F_{s}}$, set F(y)=0, y=0or1,

Currently, $\frac{dF\left(y\right)}{d_{y}}|y=0>0$, $\frac{dF\left(y\right)}{d_{y}}|y=1<$ 0, y=1 is the equilibrium point, and social organisations’ positive organisation is the evolutionary equilibrium strategy.

**Fig. 2:**
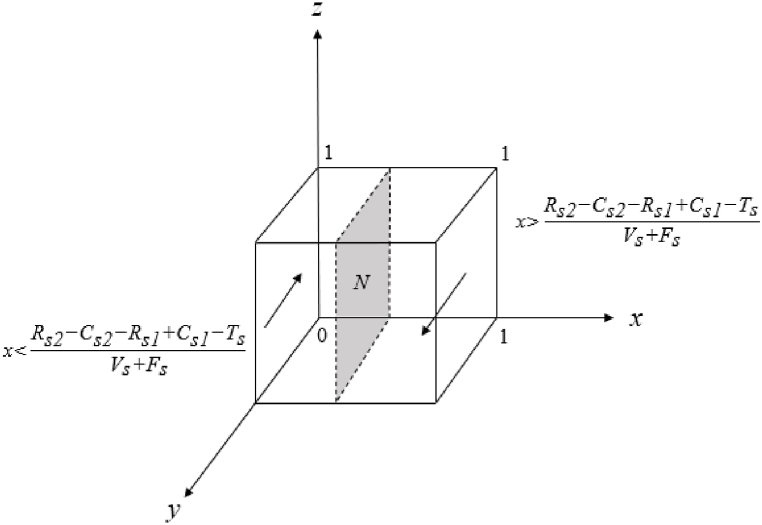
Phase diagram of the evolutionary trend of social organisations strategies.

If $x<\frac{R_{s2}‐C_{s2}‐R_{s1}+C_{s1}‐T_{s}}{V_{s}+F_{s}}$, set F(y)=0, y=0or1,

Currently, $\frac{dF\left(y\right)}{d_{x}}|y=0<0$, $\frac{dF\left(y\right)}{d_{x}}|y=1>$ 0, y=0 is the equilibrium point, and social organisations’ negative organisation is the evolutionary equilibrium strategy (Fig. 2).

In Fig. 2, social organisations’ positive organisation strategy is related to organisational beneﬁt and cost, reward and punishment mechanism and reputation loss. When other parameters are guaranteed to be constant, their stability strategy options are as follows:

① Social organisations are more likely to adapt a positive strategy when it offers high benefits at low costs, especially compared to the low benefits and high costs of negative strategies.
② Clearly defined reward and punishment mechanisms increase the likelihood of social organisations opting for positive strategies.
③ The risk of reputation loss from negative strategies makes positive strategies more appealing to social organisations.

The evolutionary game replication dynamic Equation (12) based on the public leads to the following:

If $x=\frac{R_{p2}‐C_{p2}‐R_{p1}+C_{p1}}{W_{p}}$, F(z)=0, all values of z are steady states.

If $x>\frac{R_{p2}‐C_{p2}‐R_{p1}+C_{p1}}{W_{p}}$, set F(z)=0, z=0or1,

Currently, $\frac{dF\left(z\right)}{d_{z}}|z=0>0$, $\frac{dF\left(yz\right)}{d_{z}}|z=1<$ 0, z=1 is the equilibrium point, and the public's positive participation is the evolutionary equilibrium strategy.

**Fig. 3:**
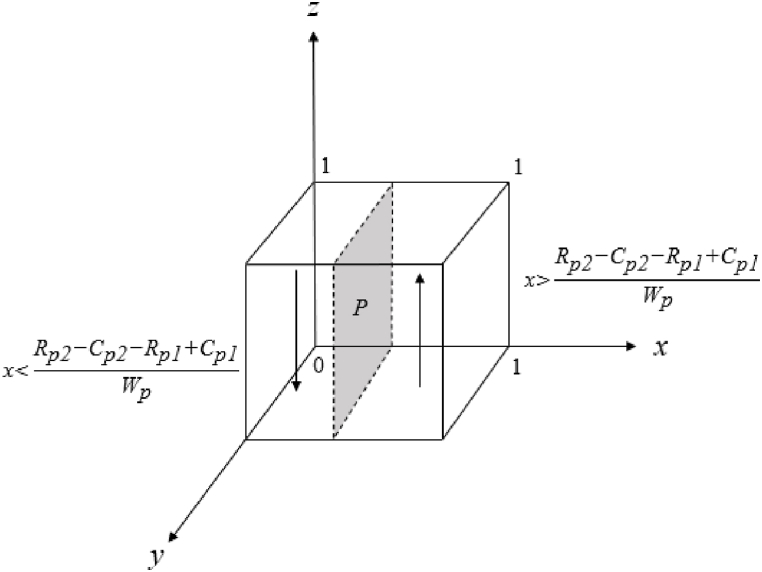
Phase diagram of the evolutionary trend of the public strategies.

If $x<\frac{R_{p2}‐C_{p2}‐R_{p1}+C_{p1}}{W_{p}}$, set F(z)=0, z=0or1,

Currently, $\frac{dF\left(z\right)}{d_{z}}|z=0<0$, $\frac{dF\left(yz\right)}{d_{z}}|z>1<$ 0, z=0 is the equilibrium point, and the public's negative participation is the evolutionary equilibrium strategy (Fig. 3).

In Fig. 3, the strategy of public participation is related to participation beneﬁt, cost and reward. When other parameters are guaranteed to be constant, the public's stability strategy options are as follows:

① The public is more likely to engage positively when such participation yields high benefits at low costs, especially in contrast to the low benefits and high costs of negative participation.
② The more rewards the local government gives to the public for positive participation, the more willing the public is to adopt the positive participation strategy.
(2) The formation of the three subjects' equilibrium strategies

Based on the replication dynamic analysis, eight special equilibrium points (0,0,0) (0,0,1) (0,1,0) (0,1,1) (1,0,0) (1,0,1) (1,1,0) (1,1,0) (1,1,1) can be obtained. Following the system stability determination method proposed by Friedman [36], the *Jacobi* matrix of the three–dimensional replication dynamic system is constructed to determine the partial stability of the equilibrium point of the three-party evolutionary game. The three-party game matrix for the emergency management of MPHE (Equation (15)) is given below.

(15) $$J=\left[\begin{array}{ccc} \left(1‐2x\right)\left[\begin{array}{c} y\left(F_{s}‐V_{s}\right)-zW_{p}\\ +R_{g1}-C_{g1}\\ -R_{g2}+C_{g2}\\ +T_{g}+F_{s} \end{array}\right] & x\left(1‐x\right)\left(F_{s}‐V_{s}\right) & x\left(1‐x\right)\left(‐W_{p}\right)\\ y\left(1‐y\right)\left(F_{s}+V_{s}\right) & \left(1‐2y\right)\left[\begin{array}{c} x\left(V_{s}+F_{s}\right)+R_{s1}\\ -C_{s1}-R_{s2}\\ +C_{s2}+T_{s} \end{array}\right] & 0\\ z\left(1‐z\right)W_{p} & 0 & \left(1‐2z\right)\left[\begin{array}{c} xW_{P}+R_{p1}\\ -C_{p1}-R_{p2}\\ +C_{p2} \end{array}\right] \end{array}\right]$$

Here, the eigenvalues of the corresponding *Jacobian* matrix can be obtained by substituting the eight special equilibrium points into the *Jacobian* matrix. The eigenvalues corresponding to each equilibrium point are shown in Table 3.

**Table 3:** Eigenvalues corresponding to each equilibrium point.

Equilibrium point	Eigenvalue		
	$\lambda_{1}$	$\lambda_{2}$	$\lambda_{3}$
*E*_1_:(0,0,0)	$R_{g1}‐C_{g1}‐R_{g2}+C_{g2}+T_{g}+F_{s}$	$R_{s1}‐C_{s1}‐R_{s2}+C_{s2}+T_{s}$	$R_{p1}‐C_{p1}‐R_{p2}+C_{p2}$
*E*_2_:(0,0,1)	$‐W_{p}+R_{g1}‐C_{g1}‐R_{g2}+C_{g2}+T_{g}+F_{s}$	$R_{s1}‐C_{s1}‐R_{s2}+C_{s2}+T_{s}$	$‐\left(R_{p1}‐C_{p1}‐R_{p2}+C_{p2}\right)$
*E*_3_:(0,1,0)	$‐V_{s}+R_{g1}‐C_{g1}‐R_{g2}+C_{g2}+T_{g}+{2F}_{s}$	$‐\left(R_{s1}‐C_{s1}‐R_{s2}+C_{s2}+T_{s}\right)$	$R_{p1}‐C_{p1}‐R_{p2}+C_{p2}$
*E*_4_:(0,1,1)	$‐V_{s}‐W_{p}+R_{g1}‐C_{g1}‐R_{g2}+C_{g2}+T_{g}+2F_{s}$	$‐\left(R_{s1}‐C_{s1}‐R_{s2}+C_{s2}+T_{s}\right)$	$‐\left(R_{p1}‐C_{p1}‐R_{p2}+C_{p2}\right)$
*E*_5_:(1,0,0)	$‐\left(R_{g1}‐C_{g1}‐R_{g2}+C_{g2}+T_{g}+F_{s}\right)$	$V_{s}+F_{s}+R_{s1}‐C_{s1}‐R_{s2}+C_{s2}+T_{s}$	$W_{p}+R_{p1}‐C_{p1}‐R_{p2}+C_{p2}$
*E*_6_:(1,0,1)	$‐\left(‐W_{p}+R_{g1}‐C_{g1}‐R_{g2}+C_{g2}+T_{g}+F_{s}\right)$	$V_{s}+F_{s}+R_{s1}‐C_{s1}‐R_{s2}+C_{s2}+T_{s}$	$‐\left(W_{p}+R_{p1}‐C_{p1}‐R_{p2}+C_{p2}\right)$
*E*_7_:(1,1,0)	$‐\left(‐V_{s}+R_{g1}‐C_{g1}‐R_{g2}+C_{g2}+T_{g}+{2F}_{s}\right)$	$‐\left(V_{s}+F_{s}+R_{s1}‐C_{s1}‐R_{s2}+C_{s2}+T_{s}\right)$	$W_{p}+R_{p1}‐C_{p1}‐R_{p2}+C_{p2}$
*E*_8_:(1,1,1)	$‐\left(‐V_{s}‐W_{p}+R_{g1}‐C_{g1}‐R_{g2}+C_{g2}+T_{g}+{2F}_{s}\right)$	$‐\left(V_{s}+F_{s}+R_{s1}‐C_{s1}‐R_{s2}+C_{s2}+T_{s}\right)$	$‐\left(W_{p}+R_{p1}‐C_{p1}‐R_{p2}+C_{p2}\right)$

From *Lyapunov's* theorem, it is known that finding the equilibrium stability point must satisfy the condition that all the eigenvalues are less than 0, which is a system stability point; if all the eigenvalues are real numbers larger than 0, it becomes a system instability point. Given that the size of trust loss, reputation loss, punishment and reward cannot be determined, the above eight points are all possible points of system stability. Table 4 presents an analysis of the stability conditions of the eight points.

**Table 4:** Stability condition corresponding to each equilibrium point.

equilibrium point	Stability condition	Serial number
$E_{1}$:(0,0,0)	$R_{g1}‐C_{g1}‐R_{g2}+C_{g2}+T_{g}+F_{s}<0$ $R_{s1}‐C_{s1}‐R_{s2}+C_{s2}+T_{s}<0$ $R_{p1}‐C_{p1}‐R_{p2}+C_{p2}<0$	①
$E_{2}$:(0,0,1)	$‐W_{p}+R_{g1}‐C_{g1}‐R_{g2}+C_{g2}+T_{g}+F_{s}<0$ $R_{s1}‐C_{s1}‐R_{s2}+C_{s2}+T_{s}<0$ $‐\left(R_{p1}‐C_{p1}‐R_{p2}+C_{p2}\right)<0$	②
$E_{3}$:(0,1,0)	$‐V_{s}+R_{g1}‐C_{g1}‐R_{g2}+C_{g2}+T_{g}+{2F}_{s}<0$ $‐\left(R_{s1}‐C_{s1}‐R_{s2}+C_{s2}+T_{s}\right)<0$ $R_{p1}‐C_{p1}‐R_{p2}+C_{p2}<0$	③
$E_{4}$:(0,1,1)	$‐V_{s}‐W_{p}+R_{g1}‐C_{g1}‐R_{g2}+C_{g2}+T_{g}+2F_{s}<0$ $‐\left(R_{s1}‐C_{s1}‐R_{s2}+C_{s2}+T_{s}\right)<0$ $‐\left(R_{p1}‐C_{p1}‐R_{p2}+C_{p2}\right)<0$	④
$E_{5}$:(1,0,0)	$‐\left(R_{g1}‐C_{g1}‐R_{g2}+C_{g2}+T_{g}+F_{s}\right)<0$ $V_{s}+{F_{s}+R}_{s1}‐C_{s1}‐R_{s2}+C_{s2}+T_{s}<0$ $W_{p}+R_{p1}‐C_{p1}‐R_{p2}+C_{p2}<0$	⑤
$E_{6}$:(1,0,1)	$‐\left(‐W_{p}+R_{g1}‐C_{g1}‐R_{g2}+C_{g2}+T_{g}+F_{s}\right)<0$ $V_{s}+F_{s}+R_{s1}‐C_{s1}‐R_{s2}+C_{s2}+T_{s}<0$ $‐\left(W_{p}+R_{p1}‐C_{p1}‐R_{p2}+C_{p2}\right)<0$	⑥
$E_{7}$:(1,1,0)	$‐\left(‐V_{s}+R_{g1}‐C_{g1}‐R_{g2}+C_{g2}+T_{g}+2F_{s}\right)<0$ $‐\left(V_{s}+F_{s}{+R}_{s1}‐C_{s1}‐R_{s2}+C_{s2}+T_{s}\right)<0$ $W_{p}+R_{p1}‐C_{p1}‐R_{p2}+C_{p2}<0$	⑦
$E_{8}$:(1,1,1)	$‐\left(‐V_{s}‐W_{p}+R_{g1}‐C_{g1}‐R_{g2}+C_{g2}+T_{g}+{2F}_{s}\right)<0$ $‐\left(V_{s}+F_{s}{+R}_{s1}‐C_{s1}‐R_{s2}+C_{s2}+T_{s}\right)<0$ $‐\left(W_{p}+R_{p1}‐C_{p1}‐R_{p2}+C_{p2}\right)<0$	⑧

E1:(0,0,0): In this scenario, the local government's negative governance strategy leads to a lack of institutional conditions and incentive mechanisms for emergency response in MPHEs. Social organisations and the public tend to adopt negative strategies without effective mobilisation. When the severity of MPHE intensifies, social organisations and the public may shift toward positive strategies. However, transitioning strategies in this context can be quite challenging.

E2:(0,0,1): In this scenario, the public adopts a passive stance, hindered by ineffective local government governance and insufficient support from social organisations. The lack of organizational leverage makes it tough for the public to independently mobilize. Given a scenario wherein fundamental governance measures are lacking, public behaviour tends to revert to E1 due to the absence of support and mobilisation. Transitioning directly to strategies like E4, E6, or E8 is relatively difficult, making it challenging to maintain stability.

E3:(0,1,0): In this scenario, without local government support or public trust, social organisations are in a passive role. Whilst the lack of local government support limits their effectiveness, social organisations can still leverage their social credibility and professional capabilities during MPHEs. Meanwhile, authoritative social organisations can influence local government through advocacy and negotiation, encouraging the shift towards E7 and E8 strategies, and facilitating effective cooperation. However, failure to take timely action can quickly lead to a shift towards the E1 strategy.

E4:(0,1,1): In this scenario, achieving stable equilibrium in the emergency game involving multiple subjects becomes challenging. The local government's negative governance strategy may interrupt active public and social organisation initiatives. Without government support, social organisations and the public might influence the government's strategy to shift through public opinion, potentially facilitating a transition towards the ideal E8 strategy.

E5:(1,0,0): In this scenario, social organisations, and the public exhibit a highly passive attitude, lacking effective participation in governance. However, as the local government supports social organisations, the latter gradually shift towards the E7 strategy. The local government can also persuade and incentivise the public to change its strategy. In this game, both subjects’ passive involvement makes cooperation difficult without the timely intervention of administrative authority.

E6:(1,0,1): This scenario depicts stable collaboration between the local government and the public, significantly enhancing MPHE emergency management. In contrast, social organisations stay passive, potentially hindered by high participation costs or flawed structures. Yet, with encouragement and policy support from the local government, these organisations may shift towards more active strategies.

E7:(1,1,0): In this scenario, the local government and social organisations reach a consensus, adopting relatively positive governance and organisational strategies for the MPHE. However, the public's passive involvement can somewhat impact the effectiveness of governance. Due to the authority and organisational advantages of the government and social organisations, they can quickly mobilize more community members, facilitating a shift towards the ideal strategies.

E8:(1,1,1): In this scenario, all three subjects easily meet the stable conditions of the game, representing the most ideal outcome for the participation of multiple subjects in emergency response. All subjects actively engage in governance, leading to a collaborative approach across society.

## 3 Simulation analysis

Based on the above replication dynamic equations and stable points analysis, MATLAB software will be used for numerical simulation and emulation to show the game process more intuitively and emphasise the influence of different variables on each subject's behaviour. Here, the probability of each subject taking different strategies is between 0 and 1. In general, there are two kinds of strategies: positive and negative. The probability above 0.5 is defined as the ‘initial positive’, and the positivity increases from 0.5 to 1, whereas the probability below 0.5 is defined as the ‘initial negative’, and the negativity increases from 0.5 to 0.

### 3.1 Simulation scenario analysis under different stable strategies

In accordance with the game model stability analysis, the strategy choices of the local government, social organisations, and the public in the emergency management of MPHEs under different stability conditions tend to vary. Therefore, the evolution process of the strategy combination must first be simulated numerically under the above eight stability conditions.

#### 3.1.1 Four strategies of negative governance by local government

**Fig. 4:**
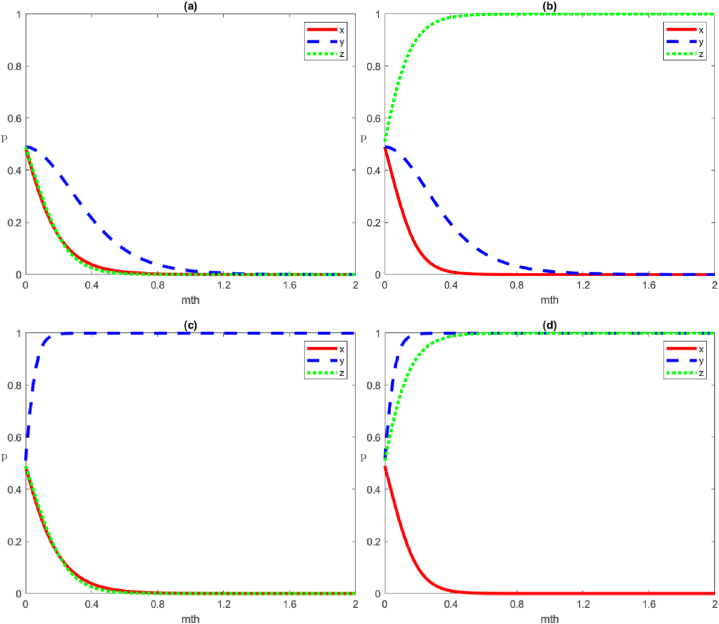
Evolutionary trajectories of game subjects under stability conditions ①,②, ③ and ④. From (a) to (d), the equilibrium points of *E*_*1*_:(0,0,0), *E*_*2*_:(0,0,1), *E*_*3*_:(0,1,0) and *E*_*4*_:(0,1,1), are represented respectively.

The four scenario simulations under the negative governance strategy by local government are shown in Fig. 4, representing the evolutionary trends of the four equilibrium points of E1:(0,0,0), E2:(0,0,1), E3:(0,1,0) and E4:(0,1,1), respectively.

Scenario 1 Evolution of the strategies of negative governance, negative organisation and negative participation by the local government, social organisations, and the public, respectively

We set the parameters as $C_{g1}=50$, $C_{g2}=20$, $R_{g1}=100$, $R_{g2}=90$, $C_{s1}=40$, $C_{s2}=30$, $R_{s1}=70$, $R_{s2}=70$, $V_{s}=5$, $F_{s}=5$, $C_{p1}=40$, $C_{p2}=20$, $R_{p1}=60$, $R_{p2}=50$, $W_{p}=5$. The evolutionary game in this scenario state must satisfy the condition of $R_{g1}‐C_{g1}‐R_{g2}+C_{g2}+T_{g}+F_{s}<0$, $R_{s1}‐C_{s1}‐R_{s2}+C_{s2}+T_{s}<0$, $R_{p1}‐C_{p1}‐R_{p2}+C_{s2}<0$. This means that the gap between benefit and cost (including trust loss, reputation loss, punishment, and reward) for all three subjects under positive strategies is smaller than that in the negative strategies. In other words, when the net profit of the positive strategy is smaller than that in the negative strategy, multiple subjects tend to adopt the negative strategy (Fig. 4(a)).

Scenario 2 Evolution of the strategies of negative governance, negative organisation and positive participation by the local government, social organisations, and the public, respectively

We set the parameters as $C_{g1}=50$, $C_{g2}=20$, $R_{g1}=100$, $R_{g2}=90$, $C_{s1}=40$, $C_{s2}=30$, $R_{s1}=70$, $R_{s2}=70$, $V_{s}=5$, $F_{s}=5$, $C_{p1}=20$, $C_{p2}=40$, $R_{p1}=50$, $R_{p2}=60$, $W_{p}=5$. Compared to Scenario 1, the parameter settings increase the net profit of the public's positive participation $\left(R_{p1}‐C_{p1}\right)$ and decrease the net profit of the negative participation $\left(R_{p2}‐C_{p2}\right)$. In this scenario, the evolution game must satisfy the stability condition $‐W_{p}{+R}_{g2}‐C_{g1}‐R_{g2}+C_{g2}+T_{g}+F_{s}<0，R_{s1}‐C_{s1}‐R_{s2}+C_{s2}+T_{s}<0，‐\left(R_{p1}‐C_{p1}‐R_{p2}+C_{p2}\right)<0$, in which the gap between the benefit and cost of the public positive participation strategy is greater than that in the negative strategy, and the public chooses the positive participation strategy. In comparison, the local government and social organisations choose the same strategies as in Scenario 1, which are the negative governance and negative organisation strategies, respectively (Fig. 4(b)).

Scenario 3 Evolution of the strategies of negative governance, positive organisation and negative participation by the local government, social organisations, and the public, respectively

We set the parameters as $C_{g1}=50$, $C_{g2}=20$, $R_{g1}=100$, $R_{g2}=90$, $C_{s1}=20$, $C_{s2}=40$, $R_{s1}=70$, $R_{s2}=70$, $V_{s}=5$, $F_{s}=5$, $C_{p1}=40$, $C_{p2}=40$, $R_{p1}=50$, $R_{p2}=60$, $W_{p}=5$. Compared to Scenario 1, the parameter settings increase the net profit of the social organisations’ positive organisation $\left(R_{s1}‐C_{s1}\right)$ and decrease the net profit of the negative organisation $\left(R_{s2}‐C_{s2}\right)$. In this scenario, the evolution game must satisfy the stability condition $F_{s}‐V_{s}+R_{g1}‐C_{g1}‐R_{g2}+C_{g2}+T_{g}+F_{s}<0，‐\left(R_{s1}‐C_{s1}‐R_{s2}+C_{s2}+T_{s}\right)<0，R_{p1}‐C_{p1}‐R_{p2}+C_{p2}<0$, in which the gap between the benefit and cost of social organisations positive organisation strategy is greater than that in the negative strategy (including the loss of trust under negative organisation strategy), and social organisations choose the positive organisation strategy. In comparison, the local government and the public choose the same strategies as in Scenario 1, which are the negative governance and negative participation strategies, respectively (Fig. 4(c)).

Scenario 4 Evolution of the strategies of negative governance, positive organisation and positive participation by local government, social organisations, and the public, respectively

We set the parameters as $C_{g1}=50$, $C_{g2}=20$, $R_{g1}=100$, $R_{g2}=90$, $C_{s1}=20$, $C_{s2}=40$, $R_{s1}=70$, $R_{s2}=70$, $V_{s}=5$, $F_{s}=5$, $C_{p1}=20$, $C_{p2}=40$, $R_{p1}=50$, $R_{p2}=60$, $W_{p}=5$. In this scenario, the condition of $F_{s}‐V_{s}‐W_{p}+R_{g1}‐C_{g1}‐R_{g2}+C_{g2}+T_{g}+F_{s}<0,‐\left(R_{s1}‐C_{s1}‐R_{s2}+C_{s2}+T_{s}\right)<0,‐\left(R_{p1}‐C_{p1}‐R_{p2}+C_{p2}\right)<0$ must be satisfied, that is, the gap between benefit and cost under the positive organisation strategy of social organisations must be greater than that in the negative organisation strategy (including the loss of trust under the negative organisation strategy), and social organisations adopt the positive organisation strategy. Furthermore, the gap between benefit and cost under the positive participation strategy of the public is greater than the negative participation strategy, and the public adopts the positive participation strategy. In contrast to social organisations, the local government tends to adopt the negative governance strategy (Fig. 4(d)).

#### 3.1.2 Four strategies of positive governance by the local government

**Fig. 5:**
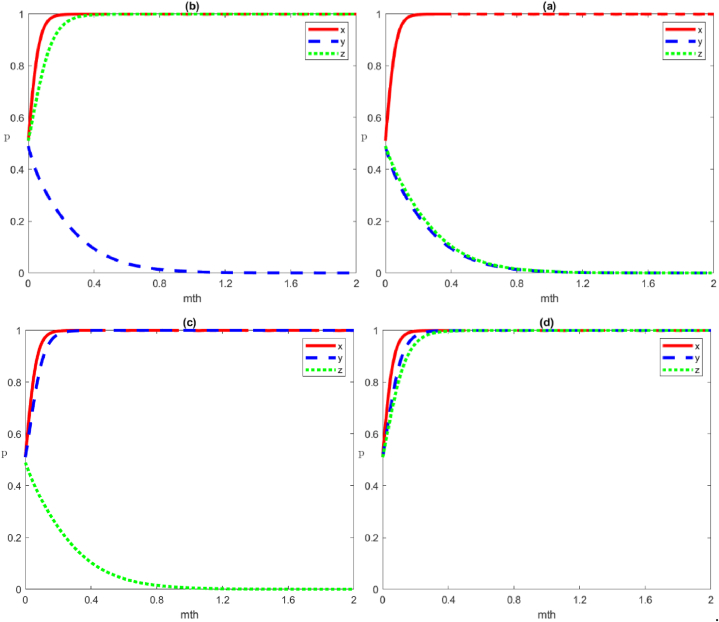
Evolutionary trajectories of game subjects under stability conditions ⑤, ⑥, ⑦ and ⑧. From (a) to (d), the equilibrium points of *E*_5_:(1,0,0), *E*_6_:(1,0,1), *E*_7_:(1,1,0) and *E*_8_:(1,1,1) are represented respectively.

The four scenario simulations under the local government's positive governance strategy are shown in Fig. 5, corresponding to the evolutionary trends of the four equilibrium points *E*_5_:(1,0,0), *E*_6_:(1,0,1), *E*_7_:(1,1,0) and *E*_8_:(1,1,1).

Scenario 5 Evolution of the strategies of positive governance, negative organisation and negative participation by the local government, social organisations, and the public, respectively.

Here, we set the parameters as $C_{g1}=20$, $C_{g2}=50$, $R_{g1}=90$, $R_{g2}=100$, $C_{s1}=50$, $C_{s2}=30$, $R_{s1}=70$, $R_{s2}=70$, $V_{s}=5$, $F_{s}=5$, $C_{p1}=40$, $C_{p2}=20$, $R_{p1}=60$, $R_{p2}=50$, $W_{p}=5$. The evolutionary game in this scenario must satisfy the condition of $‐\left(R_{g1}‐C_{g1}‐R_{g2}+C_{g2}+T_{g}+F_{s}\right)<0，V_{s}+F_{s}+R_{s1}‐C_{s1}‐R_{s2}+C_{s2}+T_{s}<0，W_{p}+R_{p1}‐C_{p1}‐R_{p2}+C_{p2}<0$, that is, the gap between benefit and cost of local government's positive governance strategy is smaller than the negative governance strategy (including punishment for social organisations under positive governance strategies, also known as the loss of trust under negative governance), and the local government adopts the positive governance strategy. In this case, the gap between the benefit and cost of social organisations' positive organisation strategy is smaller than that in the negative organisation strategy (including the reward of the positive organisation strategy and the loss of trust reputation and punishment of the negative strategy), and social organisations adopt the negative organisation strategy. Furthermore, the gap between the benefit and cost of the public's positive participation strategy is smaller than that in the negative participation strategy, and the public adopts the negative participation strategy (Fig. 5(a)).

Scenario 6 Evolution of the strategies of positive governance, negative organisation and positive participation by the local government, social organisations, and the public, respectively

We set the parameters as $C_{g1}=20$, $C_{g2}=50$, $R_{g1}=90$, $R_{g2}=100$, $C_{s1}=50$, $C_{s2}=30$, $R_{s1}=70$, $R_{s2}=70$, $V_{s}=5$, $F_{s}=5$, $C_{p1}=20$, $C_{p2}=40$, $R_{p1}=50$, $R_{p2}=60$, $W_{p}=5$. The evolutionary game in this scenario must satisfy the condition of $‐\left({‐W}_{p}+R_{g1}‐C_{g1}‐R_{g2}+C_{g2}+T_{g}+F_{s}\right)<0，V_{s}+F_{s}+R_{s1}‐C_{s1}‐R_{s2}+C_{s2}+T_{s}<0，‐\left(W_{p}+R_{p1}‐C_{p1}‐R_{p2}+C_{p2}\right)<0$, that is, the gap between benefit and cost of the local government's positive governance strategy is greater than that in the negative governance strategy (including the punishment benefit for social organisations under positive governance strategy, the reward cost for the public, and the loss of trust under negative governance strategy), and the local government adopts the positive governance strategy. In this case, the gap between the benefit and cost of the public's positive participation strategy is greater than that in the negative participation strategy (including reward for positive participation), and the public adopts the negative participation strategy. Moreover, the gap between the benefit and cost of social organisations' positive organisation strategy is smaller than the negative organisation strategy (including rewards under positive organisation strategy and the loss of trust reputation and punishment under negative organisation strategy), and social organisations adopt the negative organisation strategy (Fig. 5(b)).

Scenario 7 Evolution of the strategies of positive governance, positive organisation and negative participation by the local government, social organisations, and the public, respectively

Here, we set the parameters as $C_{g1}=20$, $C_{g2}=50$, $R_{g1}=90$, $R_{g2}=100$, $C_{s1}=30$, $C_{s2}=35$, $R_{s1}=70$, $R_{s2}=70$, $V_{s}=5$, $F_{s}=5$, $C_{p1}=40$, $C_{p2}=20$, $R_{p1}=60$, $R_{p2}=50$, $W_{p}=5$. The evolutionary game in this scenario needs to satisfy the condition of $‐\left(F_{s}‐V_{s}+R_{g1}‐C_{g1}‐R_{g2}+C_{g2}+T_{g}+F_{s}\right)<0，‐\left(V_{s}+F_{s}+R_{s1}‐C_{s1}‐R_{s2}+C_{s2}+T_{s}\right)<0$， $W_{p}+R_{p1}‐C_{p1}‐R_{p2}+C_{p2}<0$, in which the gap between benefit and cost of local government's positive governance strategy is greater than that in the negative governance strategy (including the punishment benefit for social organisations under positive governance strategy, the reward cost for the public and the loss of trust under negative governance strategy), and the local government adopts the positive governance strategy. In this case, the gap between the benefit and cost of social organisations' positive organisation strategy is greater than that in the negative organisation strategy (including reward under positive organisation strategy and the loss of trust reputation and punishment under negative organisation strategy), and social organisations adopt the positive organisation strategy. Furthermore, the gap between the benefit and cost of the public's positive participation strategy is smaller than the negative participation strategy (including reward for positive participation), and the public adopts the negative participation strategy (Fig. 5(c)).

Scenario 8 Evolution of the strategies of positive governance, positive organisation and positive participation by the local government, social organisations, and the public, respectively.

We take the parameters as $C_{g1}=20$, $C_{g2}=50$, $R_{g1}=90$, $R_{g2}=100$, $C_{s1}=30$, $C_{s2}=35$, $R_{s1}=70$, $R_{s2}=70$, $V_{s}=5$, $F_{s}=5$, $C_{p1}=40$, $C_{p2}=20$, $R_{p1}=60$, $R_{p2}=50$, $W_{p}=5$. The evolutionary game in this scenario needs to satisfy the condition of $‐\left(F_{s}‐V_{s}+R_{g1}‐C_{g1}‐R_{g2}+C_{g2}+T_{g}+F_{s}\right)<0，‐\left(V_{s}+F_{s}+R_{s1}‐C_{s1}‐R_{s2}+C_{s2}+T_{s}\right)<0$， $W_{p}+R_{p1}‐C_{p1}‐R_{p2}+C_{p2}<0$, in which the gap between benefit and cost for local government, social organisations and the public under the positive strategy is greater than negative strategy (including punishment benefit, reward cost and the loss of trust and reputation, etc.); thus, all three subjects will adopt the positive strategy (Fig. 5(d)).

### 3.2 Simulation analysis of the evolutionary impact of the initial values with different subject strategies

Analysis of the replication dynamic equations reveals that the evolutionary equilibrium of one subject is influenced by the decision proportions of others. To address the unique initial value settings for the tripartite game strategies noted in prior analyses, we will adjust these values to test the group strategy evolution, ensuring the reliability of our results. When the evolution of the three-party game reaches the strategy of *E*_8_(1,1,1), this is considered the optimal state. Therefore, this scenario is used for simulation by examining the influence of the evolution in initial values on the behaviour strategies of different subjects. First, the parameter values, which are the same as those in scenario 8, satisfy the stability condition ⑧. The impact of the change of the subject's initial value on its strategy and the evolution of the initial value of the other two subjects on their strategies are discussed in the following subsections.

#### 3.2.1 Effect of the initial value on local government strategy

**Fig. 6:**
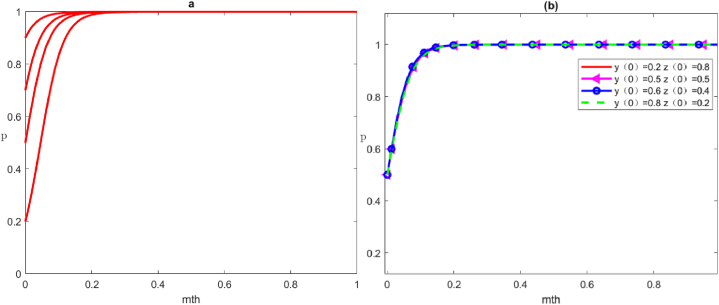
Evolutionary trajectories of the influence of the change in the initial values of the local government's strategies. (a) The initial values of the strategy of local government are set to be 0.2, 0.4, 0.6, 0.8; (b) shows the evolution of local government strategies as the initial strategy values of social organisations and public change.

The evolutionary trajectory of the influence of the change in the initial value on the local government's strategy with three game subjects is shown in Fig. 6. Specifically, Fig. 6(a) shows the evolutionary trajectory of the local government's strategy with the change of the initial value by itself. As the initial value increases, a greater emphasis on positive governance by the local government leads to a quicker and more direct evolution of the system towards x→1. In Fig. 6(b), the change (0.2→0.8) in the initial value of social organisations' strategy has almost no effect on the local government, and the two lines almost overlap. Furthermore, the change (0.2→0.8) in the initial value of social organisations strategy drives the probability of the local government to decrease in the same time, which may be due to the high degree of positive participation by the public itself. Thus, the local government can achieve the optimal state without too much intervention.

#### 3.2.2 Effect of the initial value on social organisations strategy

**Fig. 7:**
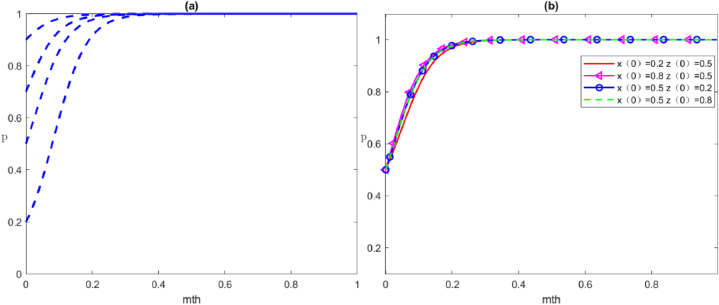
Evolutionary trajectories of the influence of the change in the initial values of social organisations' strategies. (a) The initial values of the strategy of social organisations are set to be 0.2, 0.4, 0.6, 0.8; (b) shows the evolution of social organisations' strategies as the initial strategy values of the local government and public change.

The evolutionary trajectory of the influence of the change in the initial value on social organisations' strategy with three game subjects is shown in Fig. 7. Specifically, Fig. 7(a) shows the evolutionary trajectory of the strategy with the change in the initial value by the social organisations themselves. As the initial value increases, the larger proportion of social organisations adopting positive governance strategy at the initial stage leads to a shorter and faster evolution of the system to x→1. As shown in Fig. 7(b)–as the initial strategy values of the local government and the public change, the social organisations' strategies evolve. In general, the change (0.2→0.8) in the initial value of the public strategies has almost no effect on social organisations, and the two lines almost overlap, which may be due to the small mobilisation of the public and the minimal effect of public mobilisation on the social organisations. Additionally, altering the local government's strategy initial value from 0.2 to 0.8 results in a higher likelihood of social organisations adapting correspondingly during the same period. Consequently, the initial strategic approach adopted by local governments can profoundly influence the directional evolution of social organisations' strategies.

#### 3.2.3 Effect of the initial value on the public's strategy

**Fig. 8:**
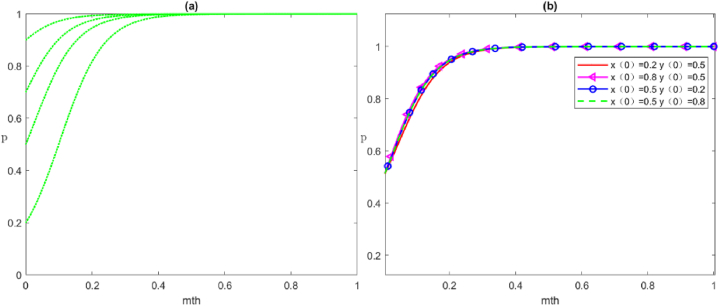
Evolutionary trajectory of the influence of the change in the initial values of the public's strategies. (a) The initial values of the strategy of social organisations are set to be 0.2, 0.4, 0.6, 0.8; (b) shows the evolution of social organisations' strategies as the initial strategy values of the local government and the public change.

The evolutionary trajectory of the influence of the change in the initial value on the public's strategy with three game subjects is shown in Fig. 8. Specifically, Fig. 8(a) shows the evolutionary trajectory of the strategies with the change in the public's initial value. An increase in the initial value, leading to a greater initial adoption of positive strategies by the public, results in a quicker and more direct evolution of the system towards x→1. The evolution of public participation strategies with the change of initial strategy values of the local government and social organisations is shown in Fig. 8(b). Generally, adjusting the initial value of social organisations' strategies from 0.2 to 0.8 scarcely impacts public participation behaviour, resulting in nearly identical outcomes. Simultaneously, adjusting the local government's strategy initial value from 0.2 to 0.8 encourages the public to similarly increase their engagement probability. Thus, the local government's initial strategy can have significant positive guidance on the strategy evolution of social organisations.

**Fig. 9:**
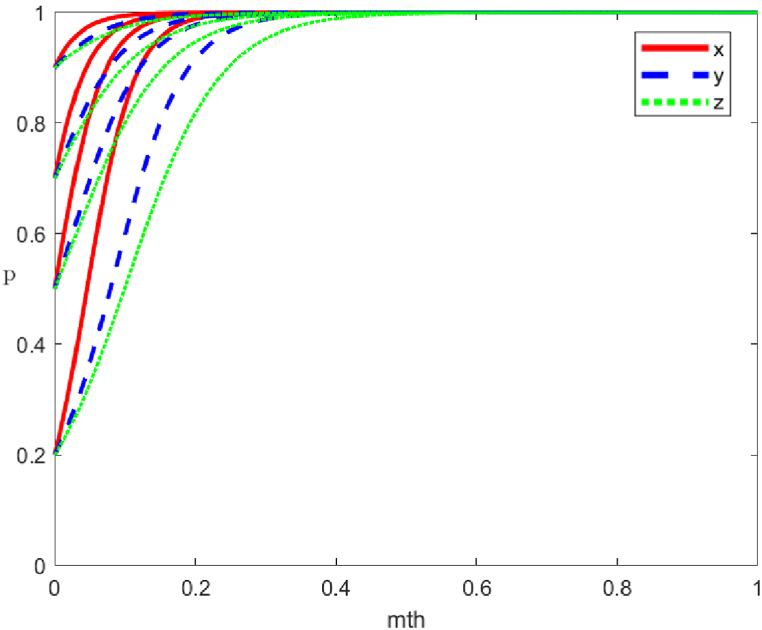
Evolutionary trajectories of each subject's initial value on the influence of participation strategy.

Based on the above changes in the initial values of the strategies with multiple subjects, the following conclusions can be drawn: (1) Each subject's initial value changes have an impact on the evolution of its strategy, in which the higher the initial value, the more positive the strategy adopted and the shorter the time required for it to evolve into the optimal strategy. In addition, as shown in Fig. 9, when the initial strategy values of the three subjects are the same, the local government has the shortest time to evolve into an optimal strategy, followed by social organisations and the public. (2) The initial value of each subject's strategy, which is the difference of the initial values of x, y and z, will have some influence on the group's strategy evolution path under the premise of satisfying the interests of each subject. However, it will not change the final strategy choice of the group, amongst which the change of the initial value of the government's strategy has the greatest influence, followed by changes in the public and social organisations.

### 3.3 Simulation analysis of the impact of the local government's reward and punishment on each subject's strategy

In the management of MPHEs, the cost–benefit of the three subjects in the governance process affects their strategy choice and leads to a scenario in which the punishment and reward strength from the local government will have an impact on the strategy choice of social organisations and the public.

In the management of MPHEs, the cost-benefit analysis of the three subjects influences their strategic decisions, resulting in a dynamic where the intensity of rewards and punishments imposed by local governments significantly shapes the strategic orientation of social organisations and the public. Therefore, scenarios *E*_6_:(1,0,1) and *E*_7_:(1,1,0) are chosen as the basis to study the trend of local government's punishment and reward intensity on the evolution of strategies adopted by social organisations and the public.

#### 3.3.1 Effects of the local government's punishment intensity on the evolutionary strategies of social organisations

**Fig. 10:**
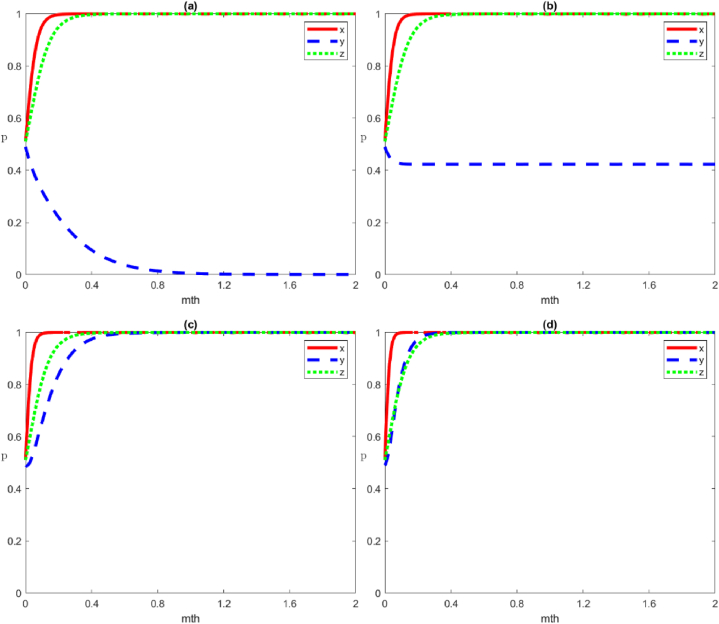
Evolutionary trajectories of the local government's punishment intensity affecting social organisations' strategies. From (a) to (d), the local government's punishment for social organisations $F_{s}=5;\;10;\;20;\;30$.

Fig. 10 corresponds to the effect of different intensities of punishments meted out by the local government on the evolutionary strategies of social organisations, assuming that other parameters are constant. The change in the local government's punishment intensity for the negative organisation of social organisations has a significant impact on the system's evolution path. As shown in Fig. 10(a), we can see the initial diagram that satisfies the conditions for the evolutionary stability of the strategies of the local government, social organisations, and the public (positive governance, negative organisation, and positive participation, respectively). Currently, the local government's punishment for social organisations $F_{s}=5$ satisfies the condition $V_{s}+F_{s}+R_{s1}‐C_{s1}‐R_{s2}+C_{s2}+T_{s}<0$. To ensure that the local government and the public parameters remain unchanged, the increase in the local government's punishment on social organisations' negative organisation strategy leads us to set $F_{s}=10$. Currently, $V_{s}+F_{s}+R_{s1}‐C_{s1}‐R_{s2}+C_{s2}+T_{s}=0$, the strategy of social organisations evolves to Fig. 10(b). Then, to control for the parameters of the local government and the public, ensuring that they remain unchanged, we continue to increase the former's punishment on social organisations' negative organisation strategy, set $F_{s}=20$, $F_{s}=30$. Currently, $V_{s}+F_{s}+R_{s1}‐C_{s1}‐R_{s2}+C_{s2}+T_{s}>0$, the strategy of social organisations evolves to Fig. 10(c) and (d). Therefore, when the local government's punishment for the negative strategy of social organisations is small, the strategy of the latter evolves towards the negative organisation. As such, the increase in government punishment for the negative organisation of social organisations accelerates the convergence rate of social organisations' evolution towards the positive organisation.

#### 3.3.2 Effects of the local government's reward strength on the evolutionary strategies of social organisations and the public

(1) Evolution of social organisations strategies

**Fig. 11:**
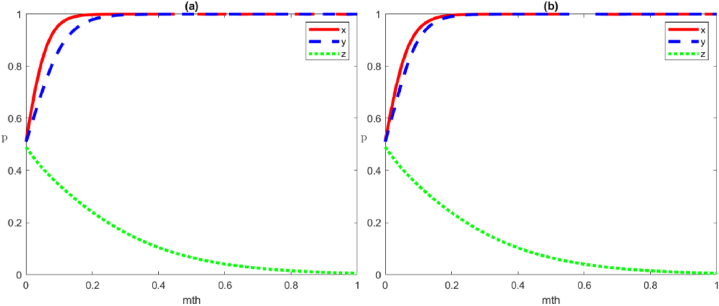
Evolutionary trajectories of local government reward strength affecting social organisations' strategies. From (a) to (b), local government's reward for social organisations $V_{s}=5;\;10$.

Fig. 11 corresponds to the effects of different rewards from the local government on social organisations' strategy evolution, assuming all other parameters are constant. Fig. 11(a) shows the initial diagram that satisfies the stability condition of the strategy evolution of the local government, social organisations, and the public (positive governance, positive organisation, and negative participation, respectively). Currently, the local government rewards $V_{s}=5$ to social organisations, and the former strengthens the reward for the latter's positive organisation and increases the reward to $V_{s}=10$. As such, the strategic evolution of social organisations accelerates their evolution towards the optimal strategy Fig. 11(b).

(2) Evolution of the public's strategies

**Fig. 12:**
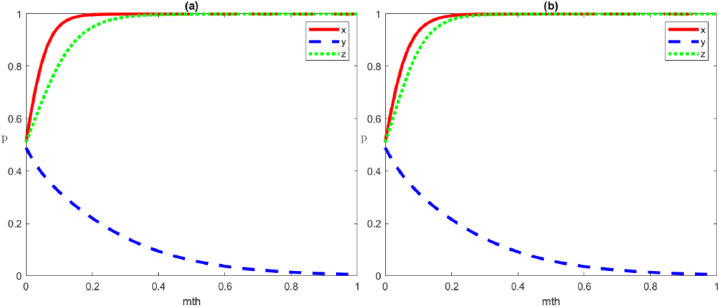
Evolutionary trajectories of local government reward strength affecting the public strategies. From (a) to (b), local government reward for the public $W_{p}=5;\;10$.

Fig. 12 Shows the effects of different rewards by the local government on the evolutionary strategy of public participation, assuming other parameters are constant. As can be seen, Fig. 12(a) shows the initial diagram that satisfies the stable condition of the evolution of the strategies by local government, social organisations, and the public (positive governance, negative organisation, and positive participation, respectively). Currently, the local government reward $W_{p}=5$ for the public's positive participation. As the local government increases the reward strength for its positive participation to $W_{p}=10$, the public's strategy evolves as shown in Fig. 12(b). Furthermore, the time to evolve to the optimal strategy of x→1 becomes shorter and faster.

From the simulation analysis of the influence of the above variables on each subject's strategy, the following conclusions can be drawn: (1) the local government acts as a regulator for the negative organisational strategy of social organisations and can promote the transformation of social organisations' strategy by appropriately increasing the punishment, and (2) the local government encourages the positive strategy of social organisations and the public whilst also promoting the transformation of both subjects' strategy to the optimal one by appropriately increasing its rewards.

## 4 Conclusion and discussion

### 4.1 Conclusion

This paper constructs a multisubject game model for the emergency management of MPHEs and explores the strategy combinations and influencing factors of multisubject games in different situations. The conclusions are as follows:

(1) **Strategy combinations of multisubject games in different situations.** The local government, social organisations, and the public form eight different strategy combinations under different stability conditions, each corresponding to different stability conditions and can be stable under the premise of meeting the stability conditions. All three subjects adopt the negative strategy, which is considered the most undesirable strategy. When all three subjects adopt the positive strategy, then multiple subjects will easily meet the stability conditions of the game, making it the most ideal game result for multiple subjects to participate. Furthermore, the emergency management of MPHEs in this state will reach the optimal effect in a short period and achieve a stable strategy. At the same time, six permutations are generated by the three strategies in the evolution of positive and negative strategies, and for each combination, the choice of different subjects is influenced by the strategies of other subjects and may evolve in a more positive or negative direction.
(2) **Stable optimal strategies for evolutionary games with multiple subjects.** The stable optimal strategy of the multisubject game is that all three game subjects adopt positive strategies to participate in the management of MPHEs. The optimal strategy is formed by two mechanisms, the first of which is the influence of benefit–cost. When the system is in the scenario of the negative governance of the three-game subjects, as the benefit of the positive strategy and the cost of negative strategy both increase, this can directly induce the three-game subjects to gradually shift to a positive governance strategy, thus forming a stable optimal strategy of the multisubject game. However, this process is relatively difficult. The second mechanism is that the local government strategy has a promoting effect. In particular, the local government plays an important role in the management of MPHEs and its governance ability affects the strategy choice of other subjects. Negative governance weakens its regulatory and mobilization capabilities, leading to a higher likelihood of negative strategies among social organisations and the public, resulting in a non-cooperative outcome. Conversely, positive governance by the local government fosters a conducive environment for the adoption of positive strategies. Even initially negative strategies from social organisations and the public may evolve positively under the local government's influence, leading to the most favourable outcomes and a stable, optimal strategy across all actors.
(3) **Factors influencing the evolutionary game strategy of multiple subjects.** First, we investigated the effect of the change in the initial value of the strategy on the game strategy. Under the condition that the parameter values are determined, the speed of the strategy evolution for local government, social organisations and the public game subjects will be influenced by the proportions with its own initial strategy choice and with the initial strategy choice of the two other subjects, although all of them will not change the final strategy decision of the game group. Second, we also examined the influence of reward and punishment on game strategy. When the local government adopts the positive strategy, keeping the parameters of other variables constant, some external forces, including the punishment and subsidy policies of local government, the penalty policies of higher authorities and the loss of reputation and trust suffered by the negative governance of local government and social organisations, can significantly influence the strategy choice of social organisations and the public.

### 4.2 Discussion

The crux of collaborative governance challenges in managing MPHEs lies in the discord between subject values and interests. Influenced by the ‘economic man’ perspective, various governance subjects seek to align with public interests but prioritize their own. Variations in these subjects' values, capabilities, and interest demands hinder effective collaboration in MPHE governance.

However, analysing interactions in MPHE management reveals potential for achieving interest balance among local government, social organisations, and the public. This can be accomplished by promoting more constructive interactions amongst the three subject groups through strategies that maximise their respective interests.

First, local governments are pivotal in MPHE management, significantly influencing social organisations and the public with their governance strategies. To optimize governance, local authorities should first bolster their capabilities and credibility through efficient, organized management and a comprehensive information disclosure system. Furthermore, enhancing punitive and incentive measures will secure more effective governance.

Second, social organisations can compensate for government failures from various angles and engage in constructive interactions with the local government. Furthermore, social organisations should aim to enhance their normal emergency response capabilities with the goal of achieving efficient participation and mobilisation, thereby fostering the development of specialised emergency social organisations.

Third, for the public, the core focus should be on safeguarding public interests by elevating their participation in the effective management of MPHEs. This will facilitate better cooperation among the public and other subjects as well as encourage the public to adopt more proactive strategies in their interactions.

This study contributes to the literature by developing a triadic game model incorporating local government, social organisations, and the public, thereby enhancing the framework of collaborative governance in MPHEs. Serving as vital conduits for resources and information, social organisations significantly improve dialogue and collaboration between local governmental and the public. Incorporating these subjects into MPHEs reinforces resource and knowledge sharing, capitalizes on their informal sway, and fosters a Pareto-efficient allocation of resources for emergency responses.

Additionally, this study's scientific analysis of the game mechanism involving local government, social organisations, and the public demonstrates that all three subjects' strategies are influenced by cost and benefit considerations. Crucially, the local government's ability to adjust the pace and direction of strategy equilibrium through its system of rewards and punishments underscores its pivotal role. This insight offers empirical support for strategic decision-making by local governments.

This research identified several limitations. Firstly, it focused on local governments, social organisations, and the public, excluding market-related actors. Future studies could use evolutionary game theory for a broader and more detailed analysis. Secondly, MATLAB was the main tool for numerical simulations, which might not fully capture real-world complexities. Future work could involve regional case studies to gather real data. Additionally, consulting experts and professionals through interviews and surveys could improve the study's impact and recommendations.

## Data availability

The authors are unable or have chosen not to specify which data has been used.

## CRediT authorship contribution statement

**Rui Nan:** Supervision, Conceptualization. **Jing Chen:** Writing – original draft, Software, Methodology. **Wenjun Zhu:** Writing – review & editing.

## Declaration of competing interest

The authors declare the following financial interests/personal relationships which may be considered as potential competing interests:Rui Nan reports financial support was provided by National Social Science Fund of China. Rui Nan reports financial support was provided by Beijing Social Science Foundation.

## References

[1] Bedford J., Farrar J., Ihekweazu C., Kang G., Koopmans M., Nkengasong J. (2019). A new twenty-first century science for effective epidemic response. Nature.

[2] Kandel N., Chungong S., Omaar A., Xing J. (2020). Health security capacities in the COVID-19 outbreak: an analysis of International Health Regulations annual report data from 182 countries. Lancet.

[3] Sun C., Hsieh Y.-H. (2010). Global analysis of an SEIR model with varying population size and vaccination. Appl. Math. Model..

[4] Basnarkov L. (2021). SEAIR Epidemic spreading model of COVID-19. Chaos. Solitons & Fractals.

[5] Xiong C., Hu S., Yang M., Luo W., Zhang L. (2020). Mobile device data reveal the dynamics in a positive relationship between human mobility and COVID-19 infections. Proc. Natl. Acad. Sci. USA.

[6] Ojo M.M., Benson T.O., Peter O.J., Goufo E.F.D. (2022). Nonlinear optimal control strategies for a mathematical model of COVID-19 and influenza co-infection. Phys. Stat. Mech. Appl..

[7] Chakraborty T., Ghosh I. (2020). Real-time forecasts and risk assessment of novel coronavirus (COVID-19) cases: a data-driven analysis. Chaos, Solit. Fractals.

[8] Zu J., Xu F., Jin T., Xiang W. (2022). Reward and Punishment Mechanism with weighting enhances cooperation in evolutionary games. Phys. Stat. Mech. Appl..

[9] Yang L., Fang X., Zhu J. (2022). Knowledge mapping analysis of public health emergency management research based on Web of Science. Front. Public Health.

[10] Huang Y., Lou X., Wang C., Chen Z. (2022). Incentive mechanism design in collaborative management of public health emergencies. Sustainability.

[11] Fang D., Pan S., Li Z., Yuan T., Jiang B., Gan D., Liu Z. (2020). Large-scale public venues as medical emergency sites in disasters: lessons from COVID-19 and the use of Fangcang shelter hospitals in Wuhan, China. BMJ Glob. Health.

[12] Kabir K.A., Tanimoto J. (2020). Evolutionary game theory modelling to represent the behavioural dynamics of economic shutdowns and shield immunity in the COVID-19 pandemic. R. Soc. Open Sci..

[13] Tian H., Liu Y., Li Y., Wu C.H., Chen B., Kraemer M.U., Dye C. (2020). An investigation of transmission control measures during the first 50 days of the COVID-19 epidemic in China. Science.

[14] Li T., Guo Y. (2022). Optimal control and cost-effectiveness analysis of a new COVID-19 model for Omicron strain. Phys. Stat. Mech. Appl..

[15] Wei J., Wang L., Yang X. (2020). Game analysis on the evolution of COVID-19 epidemic under the prevention and control measures of the government. PLoS One.

[16] Dryhurst S., Schneider C.R., Kerr J., Freeman A.L., Recchia G., Van Der Bles A.M., Van Der Linden S. (2020). Risk perceptions of COVID-19 around the world. J. Risk Res..

[17] Song S., Yao X., Wen N. (2021). What motivates Chinese consumers to avoid information about the COVID-19 pandemic?: the perspective of the stimulus-organism-response model. Inf. Process. Manag..

[18] Eikenberry S.E., Mancuso M., Iboi E., Phan T., Eikenberry K., Kuang Y., Gumel A.B. (2020). To mask or not to mask: modeling the potential for face mask use by the general public to curtail the COVID-19 pandemic. Infectious disease modelling.

[19] Wang X., Zhang N., Zhou H., Huang X., Luo R. (2023). Multi-Agent evolutionary game analysis of group panic buying in China during the COVID-19 pandemic. Mathematics.

[20] Allington D., Duffy B., Wessely S., Dhavan N., Rubin J. (2021). Health-protective behaviour, social media usage and conspiracy belief during the COVID-19 public health emergency. Psychol. Med..

[21] Fang Y., Jia J., Li J., Liu S. (2022). Analysis of public information demand during the COVID-19 pandemic based on four-stage crisis model. Frontiers in Physics.

[22] Chen Q., Min C., Zhang W., Wang G., Ma X., Evans R. (2020). Unpacking the black box: how to promote citizen engagement through government social media during the COVID-19 crisis. Comput. Hum. Behav..

[23] Bowles S., Gintis H. (2002). Social capital and community governance. Econ. J..

[24] Marston C., Renedo A., Miles S. (2020). Community participation is crucial in a pandemic. Lancet.

[25] Zhou L., Ouyang F. (2023). Innovate emergency governance mechanism of urban communities in response to major public health events: a qualitative study from multiple principals in Guangzhou, China. Front. Public Health.

[26] Agarwal P., Al Aziz R., Zhuang J. (2022). Interplay of rumor propagation and clarification on social media during crisis events-A game-theoretic approach. Eur. J. Oper. Res..

[27] Ouyang Y., Zhao H. (2022). Evolutionary game analysis of collaborative prevention and control for public health emergencies. Sustainability.

[28] Yin S., Zhang N. (2021). Prevention schemes for future pandemic cases: mathematical model and experience of interurban multi-agent COVID-19 epidemic prevention. Nonlinear Dynam..

[29] Zhou Y., Rahman M.M., Khanam R., Taylor B.R. (2022). The impact of penalty and subsidy mechanisms on the decisions of the government, businesses, and consumers during COVID-19——tripartite evolutionary game theory analysis. Operations Research Perspectives.

[30] Nan R., Wang J., Zhu W. (2022). Evolutionary game of social network for emergency mobilization (SNEM) of magnitude emergencies: evidence from China. Complexity.

[31] Fukuyama F. (2008).

[32] Kabir K.A. (2021). How evolutionary game could solve the human vaccine dilemma. Chaos, Solit. Fractals.

[33] Knack S., Keefer P. (2007). Does social capital havean economic pay off? A cross-country investigation. Q. J. Econ..

[34] Myerson R.B. (1991).

[35] Fan R., Wang Y., Lin J. (2021). Study on multi-agent evolutionary game of emergency management of public health emergencies based on dynamic rewards and punishments. Int. J. Environ. Res. Publ. Health.

[36] Friedman D. (1991). Evolutionary games in economics. Econometrica: J. Econom. Soc..

